# The role of preoperative serum thyroglobulin in the diagnosis and treatment of differentiated thyroid cancer: a systematic review and meta-analysis

**DOI:** 10.3389/fonc.2024.1426785

**Published:** 2024-12-24

**Authors:** Ying Lu, Hao Zhao, ChunHao Liu, ZiFeng Kuang, XiaoYi Li

**Affiliations:** ^1^ Department of General Surgery, Chinese Academy of Medical Sciences & Peking Union Medical College, Beijing, China; ^2^ Surgery Centre of Diabetes Mellitus, Beijing Shi ji tan Hospital Capital Medical University, Beijing, China; ^3^ Department of General Surgery, Peking Union Medical College Hospital, Chinese Academy of Medical Sciences & Peking Union Medical College, Beijing, China

**Keywords:** differentiated thyroid cancer, serum thyroglobulin, preoperative, benign and malignant nodule differentiation, lymph node metastasis, distant metastasis

## Abstract

**Background:**

Serum thyroglobulin (Tg) is a critical marker for monitoring tumor recurrence and metastasis in patients who have undergone total thyroidectomy for differentiated thyroid cancer (DTC). While the definitive role of preoperative serum Tg in DTC is not yet established, studies suggest its importance in differentiating between benign and malignant thyroid nodules with indeterminate cytology, as well as in predicting distant metastasis (DM) in patients with DTC.

**Methods:**

A thorough literature review was conducted on the use of preoperative serum Tg in differentiating between benign and malignant thyroid nodules, and in evaluating the extent of DTC lesions. Relevant studies were systematically searched in PubMed, Embase, Cochrane, Scopus, and ClinicalTrials databases. A meta-analysis was performed on studies where the ratios between serum Tg diagnostic thresholds and the upper limit of the reference range were similar.

**Results:**

Recent studies showed significantly elevated preoperative serum Tg levels in patients with DTC compared with normal individuals. However, there are inconsistencies in the serum Tg levels between patients with preoperative DTC and benign thyroid nodules across different studies. In patients with thyroid nodules who had indeterminate cytology (negative Tg antibody), the preoperative serum Tg levels were significantly higher in malignant nodules than in benign ones (meta-analysis: odds ratio: 2.59, 95% confidence intervals: 1.59–4.21, P = 0.0001). Although the meta-analysis indicated that high preoperative serum Tg is a risk factor for central lymph node metastasis in patients with DTC (meta-analysis: odds ratio: 1.68, 95% confidence interval: 1.32–2.14, P < 0.0001), some studies suggest that high preoperative serum Tg in patients with DTC does not necessarily lead to central lymph node metastasis. Furthermore, preoperative serum Tg might possess a suggestive value regarding the likelihood of DTC patients developing DM.

**Conclusion:**

Preoperative serum Tg shows promise in differentiating between benign and malignant nodules in thyroid nodule patients with indeterminate cytology. However, further research is necessary to determine its predictive significance for lymph node metastasis and DM in patients with DTC.

**Systematic review registration:**

https://www.crd.york.ac.uk/PROSPERO/#searchadvanced, identifier CRD42024472074.

## Introduction

Primary thyroid cancer, the most common endocrine malignancy, has shown a consistent increase in incidence in recent decades (1). It is broadly categorized into follicular epithelial-derived and non-follicular epithelial-derived tumors. The former can be further categorized into differentiated thyroid cancer (DTC), poorly differentiated and undifferentiated thyroid carcinoma. Among these, DTC includes papillary thyroid carcinoma (PTC), follicular thyroid carcinoma (FTC), and Hürthle cell carcinoma (2), accounting for approximately 95% of cases (3). Effective serum markers are crucial for the diagnosis and treatment of DTC. Thyroglobulin (Tg), a large protein comprising 2750 amino acids with a molecular weight of 330 kDa, is synthesized and secreted by thyroid follicular epithelial cells, predominantly located in thyroid follicular cells and follicular lumens, with a minimal amount found in the circulation (4). In patients with post-total thyroidectomy DTC (especially those undergoing ^131^I therapy), serum Tg serves as a valuable marker for monitoring tumor residue, recurrence, and metastasis, as at this stage, its only source in the bloodstream is residual or recurrent lesions (5).

As a serum marker, Tg can be easily obtained preoperatively and offers advantages in terms of convenience, speed, good repeatability, and automatic detection (6). However, the utility of preoperative Tg as a serological indicator for the diagnosis and treatment of DTC remains controversial. The 2015 American Thyroid Association guidelines do not recommend routine preoperative Tg testing for the initial evaluation of thyroid nodules due to varying degrees of Tg elevation in various thyroid diseases (5). Ultrasonography and fine-needle aspiration biopsy (FNAB) are the main methods for preoperative differentiation between benign and malignant thyroid nodules, but they have limitations in distinguishing the benign and malignant nature of follicular tumors. Nevertheless, studies have identified preoperative serum Tg levels as a risk factor for diagnosing malignant lesions in thyroid follicular tumors (7, 8). Preoperative assessment of lymph node metastasis (LNM) in patients with DTC primarily relies on ultrasound and lymph node aspiration, but their accuracy depends on the ultrasonographer’s experience (9). Additionally, the presence of distant metastasis (DM) significantly impacts the treatment and prognosis of patients with DTC. Although computed tomography scans and whole-body nuclear imaging are commonly used to evaluate DM preoperatively, they often fail to clearly define the extent and scope of metastasis (9). Some studies have suggested that preoperative serum Tg holds predictive value for LNM (10, 11) and DM (12) in patients with DTC. However, due to methodological limitations, the diagnostic value of preoperative Tg is challenging to consistently establish across different studies and samples. One major challenge lies in the detection methods for serum Tg levels, which mainly include radioimmunoassay and immunometric assay (13). The adoption of immunometric assay over radioimmunoassay is increasing due to radioimmunoassay’s characteristics such as radioactive contamination and the need for manual operation (6). Early immunometric assay methods were primarily immunoradiometric assays, but currently, the main methods are chemiluminescent immunoassays and electrochemiluminescence immunoassays. These different detection methods yield varying Tg levels, leading to poor comparability of results between different institutions and laboratories (14). This lack of comparability hampers the consolidation and comparison of research findings from different centers using different methods, impeding in-depth discussions. Additionally, serum thyroglobulin antibody (TgAb) can interfere with serum Tg testing levels, and this interference is not directly proportional to the quantity of TgAb in the specimen. Even small amounts of antibodies can significantly affect test results (15). Patients with DTC who are TgAb positive (exceeding the upper limit of the reference range) have TgAb levels twice as high as those of normal individuals (14). Therefore, interference of serum Tg by TgAb is more common in patients with DTC. Properly including patients and excluding interference from TgAb are essential to obtain reliable research conclusions.

Considering these challenges, this paper aims to conduct a systematic review and meta-analysis of relevant literature by aggregating as much research data as possible and using appropriate methods. The objective is to assess the utility of preoperative serum Tg in distinguishing between benign and malignant thyroid nodules and predicting the severity of disease in patients with DTC. This research aims to provide valuable insights into the clinical diagnosis and treatment of DTC before surgery.

## Methods

### Search strategy

This review has been registered with PROSPERO (Registration No. CRD42024472074) and adheres to the PRISMA (Preferred Reporting Items for Systematic Reviews and Meta-Analyses) guidelines for reporting systematic reviews and meta-analyses (16, 17). The PubMed, Embase, Cochrane, Scopus, and ClinicalTrials databases were searched using a combination of subject headings and text words: (“differentiated thyroid cancer” OR “Papillary Thyroid Cancer” OR “Follicular Thyroid cancer” OR “Hürthle cell cancer”) AND “thyroglobulin” AND “preoperative” (detailed search strategy in **Appendix 1**). The search timeframe extended from the creation of the databases to January 20, 2024.

### Study selection

The study included randomized controlled trials and retrospective and prospective observational cohort studies that evaluated the relationship between preoperative serum Tg levels and the diagnosis and treatment of DTC. The inclusion criteria were as follows: patients diagnosed with DTC based on postoperative paraffin pathology, studies investigating at least one aspect of preoperative serum Tg level in the diagnosis or disease severity of DTC, and studies published in English. All retrieved literature was imported into Rayyan (http://rayyan.qcri.org), and after removing duplicates, irrelevant studies were excluded after reviewing titles and abstracts. Full-text articles were then reviewed, and studies containing non-DTC (e.g., medullary thyroid carcinoma or anaplastic thyroid cancer), non-serum Tg sources (such as aspirates, sputum, and thyroid tissue), postoperative Tg, non-English articles, posters, reviews, case reports, and letters were excluded. In cases of multiple publications from the same institution, only the latest publication was included. Literature screening was independently done by one team member and reviewed by another member. Any discrepancies in literature screening and data extraction were resolved through discussion with a third member.

### Data extraction

Data were extracted from eligible studies using a standardized table according to the established inclusion and exclusion criteria. The extracted data included study characteristics (such as first author, publication year, country, and histologic types), the number of cases in the observation and control groups, Tg levels in each group, Tg cutoff values, the fourfold table or odds ratio (OR), Tg detection methods, Tg reference ranges (for studies that did not report reference ranges, reference ranges were obtained by searching for the manufacturer’s name of the reagents), whether patients with TgAb positivity were excluded, and the main study results.

### Quality assessment

The Newcastle–Ottawa Scale (NOS) was used to evaluate the quality of the studies included in the meta-analysis (details in **Supplementary Table S1** and **Supplementary Figure S1**). The assessment criteria included the following: Selection (representativeness of the study population, selection of the study population, representativeness of the control group, selection of the control group; 4 points); comparability (similarity between the study and control groups, consideration of confounding factors; 2 points); outcome (accuracy of outcome assessment methods, consistency of measurement methods between the study and control groups, rate of non-response for outcome indicators; 3 points). The total NOS score is 9 points, with scores of 7–9 indicating high-quality studies, 4–6 indicating moderate-quality studies, and <4 indicating low-quality studies.

### Statistical analysis

A meta-analysis was conducted on studies where the ratios between serum Tg diagnostic threshold (Tg levels in differentiating outcome indicators) and the upper limit of the reference range were similar. Log OR and standard errors were calculated based on the reported OR for each study. The inverse variance method was used to combine effect sizes in RevMan 5.4 software (Cochrane Collaboration). All risk estimates were calculated with their corresponding 95% confidence intervals (CI). A P-value of <0.05 was considered statistically significant. Heterogeneity among included studies was assessed using the Cochran Q statistics and the I^2^ statistics. A fixed-effects model was used if the P-value from the heterogeneity test was >0.10 or ≤0.10 but I^2^ was ≤50%; otherwise, a random-effects model was used. I^2^ ≥ 50% was considered to indicate heterogeneity among the studies, and I^2^ ≥ 75% indicated substantial heterogeneity (18). Sensitivity analysis was performed using the trim-and-fill method in STATA 15.1 (Computer Resource Center, USA).

### Literature review

**Figure 1:**
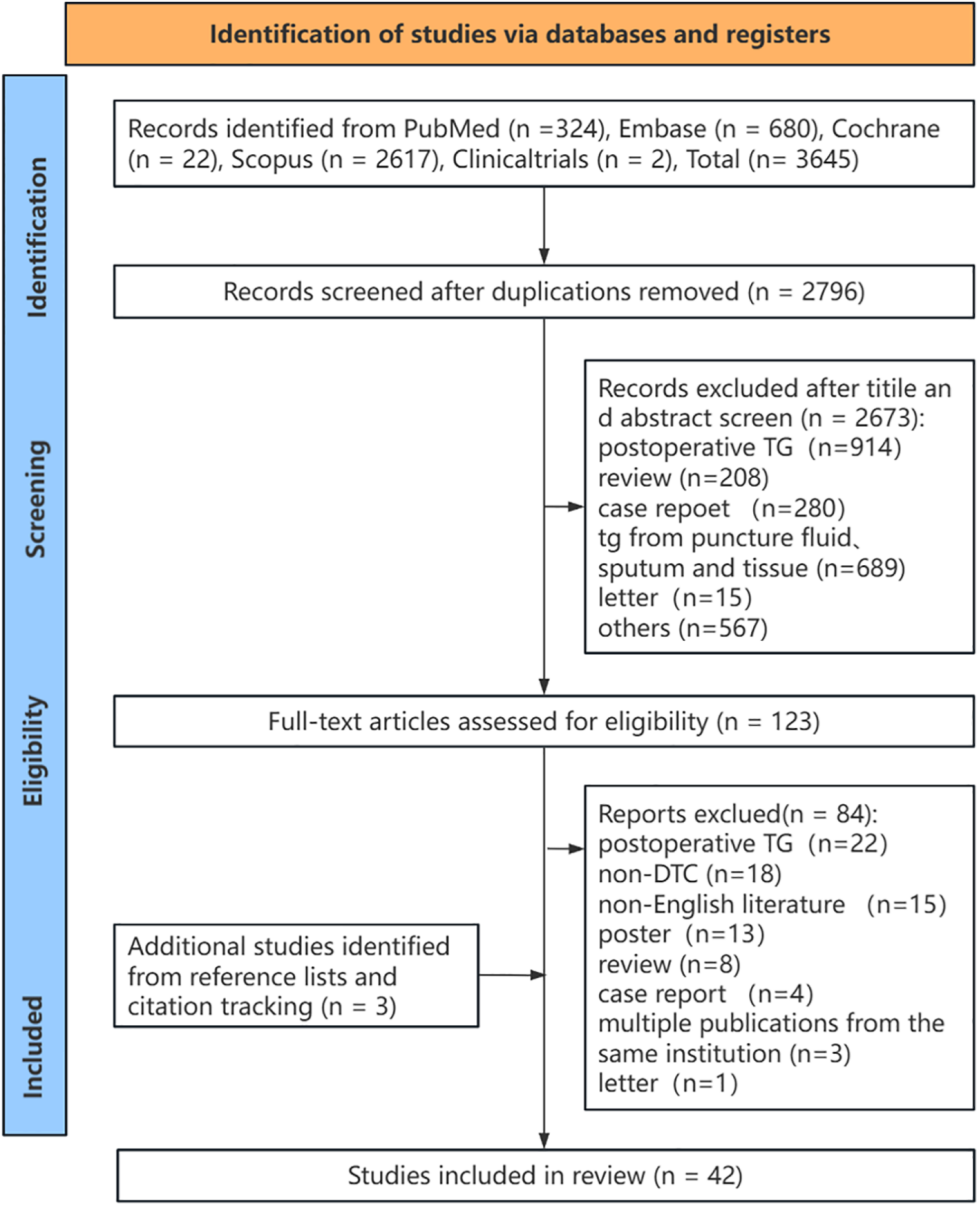
The study selection process (PRISMA flowchart). TG, thyroglobulin; DTC, differentiated thyroid cancer.

According to the search strategy, 3,645 articles were initially identified. After removing 849 duplicate articles and screening titles and abstracts, 2,673 articles were excluded. This process left 123 articles eligible for full-text retrieval. After applying the inclusion and exclusion criteria, 84 articles were excluded during the full-text assessment. Additionally, 3 articles were included through reference and citation tracking. Finally, 42 articles were deemed suitable for inclusion in the systematic review (**Figure 1**).

### The role of preoperative serum tg in discriminating thyroid nodules between benign and malignancy

Preoperative categorization of thyroid nodules as benign or malignant is crucial for treatment selection. Despite approximately 70% of individuals detecting thyroid nodules through ultrasound, even highly suspicious nodules (Thyroid Imaging Reporting and Data System class 5) carry only a 35% risk of malignancy (19). FNAB is commonly used to differentiate between benign and malignant thyroid nodules; however, 20%–30% of nodules still require further surgical intervention for definitive pathology assessment after FNAB. Preoperative serum Tg levels, a conventional serological marker, offer convenience, speed, and reliable repeatability. However, the value of serum Tg level in distinguishing between benign and malignant thyroid nodules preoperatively has not been definitively confirmed. Nevertheless, numerous studies have explored the utility of preoperative Tg levels in this regard across various populations.

In studies (All excluded TgAb positivity) focusing on healthy controls and patients with preoperative DTC, Rinaldi et al. (20) published a study in JNCI involving a prospective cohort of over 520,000 cases, excluding TgAb-positive individuals. They included 357 patients with DTC (262 PTCs, 58 FTCs, and 37 with unspecified pathology) and matched 767 healthy controls (1:3 for males and females, 1:2 matching). The findings indicated significantly elevated median preoperative serum Tg levels in both male and female patients with DTC compared with normal controls (female: 32.88 vs. 9.91 ng/mL, P < 0.001; male: 46.52 vs. 8.97 ng/mL, P < 0.001). Multivariate analysis, accounting for height and weight, revealed that with increasing preoperative serum Tg levels, the risk of diagnosing DTC increased (OR: 2.28, 95% CI: 1.93–2.71). The stratified analysis also indicated that compared with healthy controls, the risk of diagnosing PTC gradually increased with rising preoperative serum Tg levels (OR: 2.10), while the risk for FTC was even higher (OR: 4.45). Another study (21) exploring the relationship between various thyroid hormone levels and DTC incidence found that high serum Tg levels (>30 μg/L) were a risk factor for diagnosing DTC (OR: 7.0).

These studies highlight variations in the serum Tg levels between normal individuals and patients with preoperative DTC. However, the results reported by different studies regarding disparities in the serum Tg levels between benign thyroid lesions and preoperative DTC are conflicting. A substantial study by Zhang et al. involving 571 cases of benign nodules and 1519 DTCs found that patients with preoperative DTC exhibited significantly lower serum Tg levels than those with benign thyroid nodules (15.7 vs. 52.1 ng/mL, P < 0.001) (22). Moreover, the multifactorial analysis demonstrated that, as preoperative serum Tg levels decreased, the risk of diagnosing DTC increased (OR: 0.994, 95% CI: 0.991–0.998). Nevertheless, preoperative serum Tg level was not identified as an independent risk factor for diagnosing DTC. Other studies by Aydogu (23), Kang (24), and Patel (25) reached similar conclusions. Among them, Aydogu (including 517 benign nodules and 402 DTCs) revealed that the serum Tg levels were significantly higher in those with benign nodules than in patients with DTC (85.0 vs. 27.15 ng/mL; P < 0.001). However, some studies reached contradictory conclusions (26–29). Among them, the study by the Melik team (61 benign nodules and 142 PTCs) found higher serum Tg levels in patients with PTC than in those with benign nodules (105.05 vs. 76.8 ng/mL, P < 0.05). Most of these studies did not exclude patients positive for TgAb, making their conclusions debatable. In two small-sample studies that excluded TgAb-positive individuals, Guarino’s study (30) (71 benign nodules and 34 PTCs) highlighted significantly higher average serum Tg levels in patients with PTC than in those with benign nodules (282.0 vs. 85.1 ng/mL, P = 0.05). However, 80% overlap existed between the serum Tg levels of PTC and benign nodule patients, indicating the difficulty in differentiating between thyroid benign diseases and DTC based solely on preoperative serum Tg levels. Moreover, in the other study by Rigbi’s (31), no significant difference was found in the serum Tg levels between these two groups. Overall, these studies are few and have limited sample sizes, warranting further larger, well-designed studies to clarify whether preoperative serum Tg levels can differentiate between thyroid benign diseases and DTC.

Furthermore, some studies have included both normal individuals, benign thyroid patients, and patients with DTC. Among them, the study conducted by Jin (32) et al. (including 500 PTCs, 376 benign thyroid nodules, and 125 healthy controls after excluding TgAb-positive patients) found that patients with preoperative DTC exhibited significantly elevated serum Tg levels compared with those with benign nodules (42.87 vs. 33.13 ng/mL, P < 0.05). They also had significantly higher levels compared with healthy controls (42.87 vs. 14.90 ng/mL, P < 0.05), whereas no notable difference was observed in the serum Tg levels between patients with benign nodules and healthy controls. Multifactorial analysis comparing non-PTC patients (benign nodules and healthy controls) revealed that the higher the preoperative serum Tg levels, the greater the risk of diagnosing PTC (OR: 1.007, 95% CI: 1.004–1.009). Meng et al.’s study (33) also reached comparable conclusions (Not excluded TgAb positivity). However, the study by Gerfo (34) including a small number of cases did not show differences in the preoperative serum Tg levels between patients with preoperative DTC, patients with non-toxic thyroid adenomas, and healthy controls (Excluded TgAb positivity). In the aforementioned studies, the control groups were not finely matched, and the number of control cases was limited (particularly healthy controls), thus the credibility of their conclusions is limited. However, all studies consistently showed that the serum Tg levels in the healthy control remained at relatively low levels.

In conclusion, due to the insufficient number of studies, limited sample sizes, and design flaws (such as the lack of strict matching in controls and failure to exclude TgAb-positive individuals), drawing definitive conclusions regarding differences in serum Tg levels among normal individuals, patients with benign thyroid lesions, and patients with DTC remains challenging. Interestingly, in a study by Rinaldi et al. (20), the stratified analysis revealed a significant increase in the preoperative serum Tg levels for FTC, which was difficult to diagnose preoperatively through FNAB. Several studies have explored the diagnostic utility of preoperative serum Tg level in indeterminate thyroid nodules, such as FTC or follicular thyroid adenoma (FTA).

Besic et al.’s study (35), which included 288 patients with follicular tumors as seen via FNAB (postoperative diagnosis: 199 benign lesions, 24 FTCs, 3 Hürthle cell carcinomas, and 62 PTCs), found that 300 ng/mL was the optimal diagnostic cutoff value for serum Tg level to distinguish between benign and malignant lesions. Among patients with preoperative serum Tg levels of >300 ng/mL, a greater percentage were diagnosed with malignant nodules (57.30% vs. 39.20%, P = 0.004). Multifactorial analysis showed that preoperative serum Tg levels of >300 ng/mL were associated with an increased risk of postoperative malignant diagnosis in this population (OR: 1.93, 95% CI: 1.12–3.32). Similarly, Kim et al.’s study (7), which examined 198 cases considering follicular lesions on FNAB, reached similar conclusions. A study (36) with 279 Hürthle cell tumors on FNAB found that preoperative serum Tg levels of >1000 ng/mL were a risk factor for postoperative malignant diagnosis in these nodules (OR: 2.11, 95% CI: 1.08–4.15). Due to variations in serum Tg testing methods and reference value ranges in such studies, it is difficult to establish a universally applicable diagnostic threshold for Tg.

**Figure 2:**
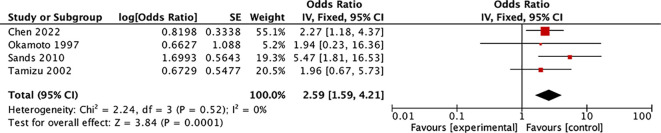
Forest plot: Meta-analysis of preoperative serum Tg level for discriminating unclear thyroid nodules on fine-needle aspiration biopsy.

In this systematic review, the ratio of the serum Tg diagnostic threshold to the upper limit of the normal range was used to standardize the influence of different testing methods. Four studies with similar ratios (range: 1–1.14) (8, 37–39) were selected for meta-analysis. The results revealed that among 539 thyroid nodules diagnosed as Bethesda III–IV preoperatively (with postoperative diagnoses of 406 benign cases and 133 malignant cases), patients with preoperative serum Tg levels exceeding the upper limit of the normal value had a significantly higher risk of being diagnosed with malignant lesions. (OR: 2.59, 95% CI: 1.59–4.21, P = 0.0001) (**Figure 2**, **Supplementary Figure S2**: funnel plot). Among these studies, Chen et al.’s study (8) demonstrated through multifactorial analysis that compared with patients with FTA, patients with preoperative serum Tg levels >434.60 ng/mL had an increased risk of being diagnosed with FTC (OR: 3.654, 95% CI: 1.528–8.738). Two additional studies from Zimmy and Lee have likewise reached similar inferences. Notwithstanding the fact that these studies did not rule out positive TgAb, the percentages of positive TgAb within the studies were considerably low, specifically 1.6% and 1.2% respectively (40, 41). However, some studies have reported conclusions that are inconsistent with the above-mentioned findings (31, 42–45). Among them, Kobayashi et al. (42) conducted a study focusing on the preoperative diagnosis of follicular tumors (811 benign nodules, 109 FTCs) and found that compared with patients whose preoperative serum Tg levels were >1000 ng/mL, patients whose preoperative serum Tg levels were ≤1000 ng/mL had a heightened risk of diagnosing FTC (OR: 1.72, 95% CI: 1.07–2.77). However, compared with the meta-analysis and studies with similar conclusions (7, 8, 35–39), most subsequent studies did not exclude TgAb-positive patients, leading to decreased credibility of their results.

In the remaining studies investigating preoperative Tg levels and their correlation with DTC differentiation, some findings stand out. Youn et al.’s study (46) uncovered a positive association between the serum Tg levels and nodule size, as well as between the serum Tg levels and thyroid volume. Another study (47) noted that upon reevaluation of patients diagnosed with follicular-variant PTC, those diagnosed with non-invasive follicular thyroid neoplasm (NIFTP) with papillary-like nuclear features exhibited lower preoperative Tg levels than those with non-NIFTP (25.55 vs. 76.06 μg/L, P = 0.0104). However, due to the limited number of studies and cases, further research is warranted to corroborate these findings.

### Predictive role of preoperative serum Tg in LNM and DM among patients with DTC

**Figure 3:**
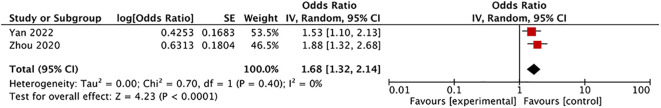
Forest plot: Meta-analysis of preoperative serum thyroglobulin level in predicting central lymph node metastasis in differentiated thyroid carcinoma patients.

Evaluating LNM and DM preoperatively in patients with DTC is crucial for assessing disease severity, prognosis, and determining treatment strategies. Recent investigations have explored the predictive potential of preoperative serum Tg for LNM and DM in DTC. In studies exploring preoperative serum Tg’s predictive role for central lymph node metastasis (CLNM) in DTC, Liu et al.’s study (48) (225 CLNM cases and 821 non-CLNM cases) found that the average preoperative serum Tg levels were lower in patients with PTC and CLNM than in those with PTC without CLNM (36.53 vs. 67.41 ng/mL, P = 0.02). Multifactorial analysis indicated that lower preoperative serum Tg levels correlated with a higher risk of diagnosing CLNM in patients with PTC (OR: 0.997). Nevertheless, this study did not exclude TgAb-positive patients, and the OR approached 1, raising doubts about the reliability of its conclusions. Conversely, two other studies (49, 50) with more robust data reached different conclusions (ratios of the Tg diagnostic threshold to the upper limit of the serum Tg normal value were 1). Their meta-analysis revealed that patients with PTC with preoperative serum Tg levels surpassing the upper limit of the normal range had a higher risk of CLNM (OR: 1.68, 95% CI: 1.32–2.14, P < 0.0001) (**Figure 3**, **Supplementary Figure S3**: funnel plot). Furthermore, multifactorial analysis in these studies suggested that higher preoperative serum Tg levels correlated with an increased risk of diagnosing CLNM in PTC. However, there are few studies to address this issue, and none have excluded patients with positive TgAb, underscoring the need for further investigation into the predictive role of preoperative serum Tg in DTC with CLNM.

Moreover, several studies have investigated the predictive capability of preoperative serum Tg levels for CLNM and lateral lymph node metastasis (LLNM) in patients with DTC. One such study by Chang et al. (10) involved 993 TgAb-negative PTC cases, including 342 CLNM cases and 104 LLNM cases. Their findings indicated that preoperative serum Tg levels of ≥31.65 mg/L in PTC were associated with a higher proportion of CLNM (OR: 3.66), while levels of ≥30.175 mg/L were associated with a higher proportion of LLNM (OR: 3.66). Multifactorial analysis further revealed that preoperative high serum Tg (≥31.65 mg/L) independently predicted CLNM in patients with PTC (OR: 1.88, 95% CI: 1.32–2.68), and high serum Tg (≥30.175 mg/L) independently predicted LLNM in patients with PTC (OR: 2.71, 95% CI:1.63–4.56). In a large sample study that has excluded TgAb positivity by Kim et al. (51), which included 4029 DTC cases, 334 of which had ipsilateral LLNM and 103 had contralateral LLNM, preoperative Tg levels higher than 13.15 ng/mL predicted ipsilateral LLNM in DTC (sensitivity 57.0%, specificity 60.6%), while levels higher than 30.05 ng/mL predicted contralateral LLNM in DTC (sensitivity 56.6%, specificity 81.4%). These findings were corroborated by a study (Not excluded TgAb positivity) conducted by Zou et al. (52), which also suggested that as preoperative serum Tg levels increased, the risk of diagnosing ipsilateral LLNM in DTC increased (OR: 2.668, 95% CI: 1.59–4.475).

However, the current body of research on the correlation between preoperative serum Tg levels and LNM in DTC is limited both in terms of quantity and sample size. Based on the two studies that excluded TgAb, it was found that the Tg levels of DTC patients with LNM were often higher. Nevertheless, many studies have failed to exclude patients with positive TgAb, and consensus on the most effective methods for validating this relationship remains elusive. Additionally, establishing an appropriate diagnostic threshold for serum Tg to distinguish the presence of LNM in DTC necessitates further investigation. Moreover, two studies (53, 54) suggesting a correlation between high Tg levels and metastasis to the lymph nodes behind the right recurrent laryngeal nerve or skip metastasis of lymph nodes warrants validation due to similar methodological concerns.

Diagnosing DM in patients with DTC presents a significant challenge. However, patients with DTC with DM typically exhibit a higher systemic tumor burden. Several studies have explored whether preoperative Tg levels hold value in indicating DM in patients with DTC. For instance, small-sample studies conducted by Edmonds (55) and Gerfo (34) demonstrated that patients with DM were likely to have higher preoperative Tg levels. Nevertheless, it is worth noting that the preoperative serum Tg in certain patients with benign thyroid nodules can also be abnormally elevated. Specifically, the proportions of patients with Tg levels surpassing 400 ng/mL and 500 ng/mL can amount to 3.64% (2/55) (55) and 8.11% (6/74) (34) respectively. However, small-sample studies by Guarino (30) and Patell (56) did not find a correlation between preoperative serum Tg and DM in patients with DTC. In contrast, a study by Kim et al. (51), which included 4029 patients with DTC (44 with DM), suggested that preoperative Tg levels exceeding 62.9 ng/mL could predict DM with a sensitivity of 85.0% and specificity of 90.6%. Similarly, Yamashita et al.’s study (57), focusing on 82 FTCs (10 with DM), showed that patients with DM had significantly elevated preoperative serum Tg levels compared with those without DM (28255 vs. 3414 ng/mL, P = 0.002), but the logistic regression analysis indicated that Tg was incapable of differentiating FTC patients with DM from those without. To sum up, the number of DM patients in these types of studies is rather limited and the analysis remains inadequate, and the reliability of the conclusions is insufficient, necessitating further accumulation of data.

In summary, the diagnostic value of preoperative serum Tg for DTC remains uncertain. However, it has shown promise in distinguishing between benign and malignant thyroid nodules in patients with indeterminate FNAB results. Further research is needed to determine whether preoperative Tg can reliably predict LNM and DM in patients with DTC. Although some studies have explored the correlation between preoperative serum Tg and the prognosis and recurrence of DTC (58, 59), the lack of sufficient and rigorous research has hindered definitive conclusions.

## Data Availability

The original contributions presented in the study are included in the article/**Supplementary Material**. Further inquiries can be directed to the corresponding author.

## Supplementary material

Supplementary Figure 1-1 Risk of bias graph: Meta-analysis of the study on the relationship between preoperative serum thyroglobulin level and indeterminate thyroid nodules.

Supplementary Figure 1-2 Risk of bias summary: Meta-analysis of the study on the relationship between preoperative serum thyroglobulin level and indeterminate thyroid nodules.

Supplementary Figure 1-3 Risk of bias graph: Meta-analysis of study on the relationship between the preoperative serum thyroglobulin level and central lymph node metastasis of differentiated thyroid cancer.

Supplementary Figure 1-4 Risk of bias summary: Meta-analysis of study on the relationship between the preoperative serum thyroglobulin level and central lymph node metastasis of differentiated thyroid cancer.

Supplementary Figure 2-1 Funnel Plot: Meta-analysis of the study on the relationship between preoperative serum thyroglobulin level and indeterminate thyroid nodules.

Supplementary Figure 2-2 Funnel Plot: Meta-analysis of the study on the correlation between preoperative serum thyroglobulin level and indeterminate thyroid nodules (drawn by using the trim-and-fill method).

Supplementary Figure 3-1 Funnel Plot: Meta-analysis of study on the relationship between the preoperative serum thyroglobulin level and central lymph node metastasis of differentiated thyroid cancer.

Supplementary Figure 3-2 Funnel Plot: Meta-analysis of study on the relationship between the preoperative serum thyroglobulin level and central lymph node metastasis of differentiated thyroid cancer (drawn by using the trim-and-fill method).
