# Supplementary figures and images for: The role of preoperative serum thyroglobulin in the diagnosis and treatment of differentiated thyroid cancer: a systematic review and meta-analysis
Source: Front Oncol. 2024 Dec 24;14:1426785. doi: 10.3389/fonc.2024.1426785 (PMC11703863; doi:10.3389/fonc.2024.1426785)

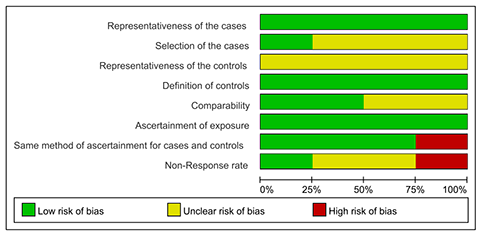

Supplement: Supplementary Figure 1-1 — Risk of bias graph: Meta-analysis of the study on the relationship between preoperative serum thyroglobulin level and indeterminate thyroid nodules. [file Image1.tif]

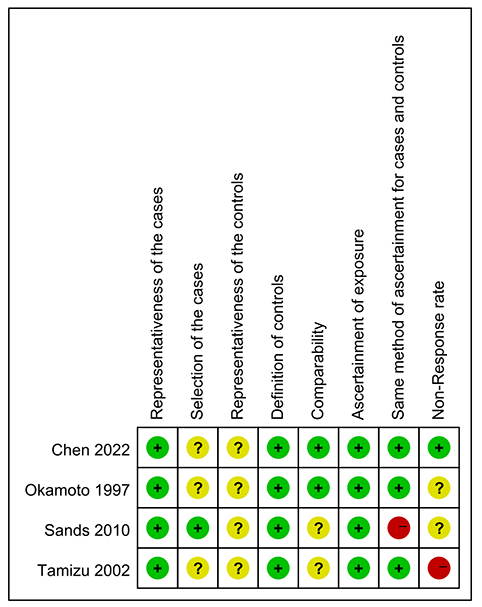

Supplement: Supplementary Figure 1-2 — Risk of bias summary: Meta-analysis of the study on the relationship between preoperative serum thyroglobulin level and indeterminate thyroid nodules. [file Image2.tif]

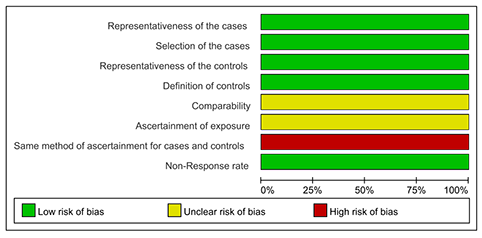

Supplement: Supplementary Figure 1-3 — Risk of bias graph: Meta-analysis of study on the relationship between the preoperative serum thyroglobulin level and central lymph node metastasis of differentiated thyroid cancer. [file Image3.tif]

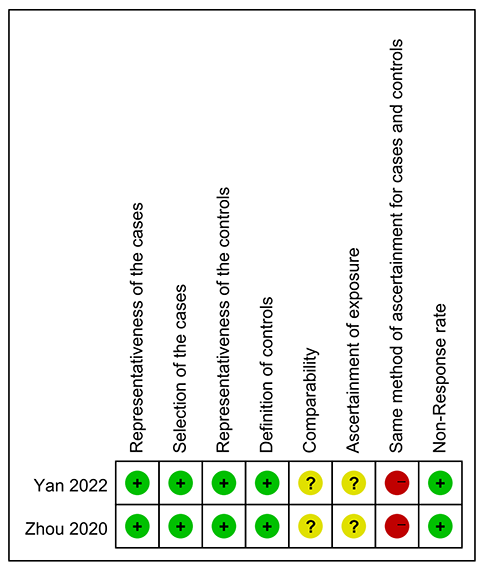

Supplement: Supplementary Figure 1-4 — Risk of bias summary: Meta-analysis of study on the relationship between the preoperative serum thyroglobulin level and central lymph node metastasis of differentiated thyroid cancer. [file Image4.tif]

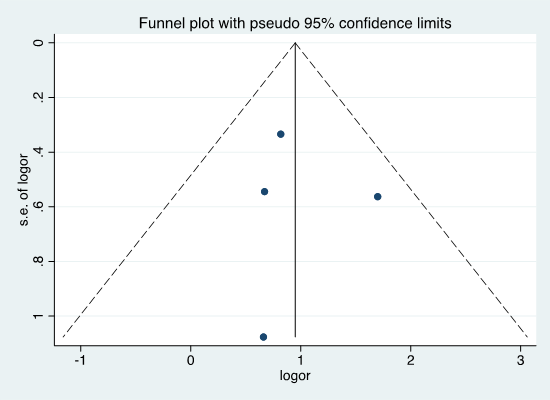

Supplement: Supplementary Figure 2-1 — Funnel Plot: Meta-analysis of the study on the relationship between preoperative serum thyroglobulin level and indeterminate thyroid nodules. [file Image5.tif]

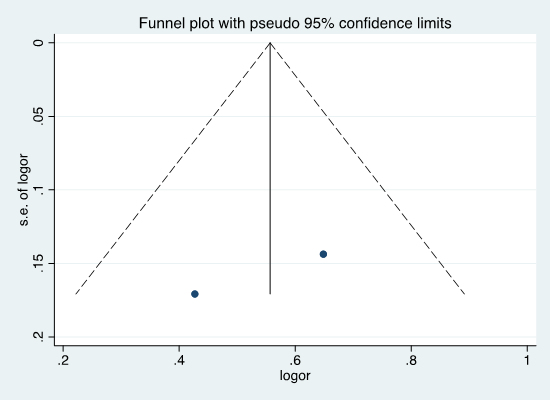

Supplement: Supplementary Figure 3-1 — Funnel Plot: Meta-analysis of study on the relationship between the preoperative serum thyroglobulin level and central lymph node metastasis of differentiated thyroid cancer. [file Image7.tif]
